# A case of intra-vaginal intrauterine testicular torsion

**DOI:** 10.1515/crpm-2022-0013

**Published:** 2022-12-20

**Authors:** Murad Habib, Muhammad Bin Amjad, Mansoor Ahmed

**Affiliations:** Department of Paediatric surgery, The Children’s hospital, Pakistan Institute of Medical Sciences, Islamabad, Pakistan; Islamabad Medical and Dental College, Islamabad, Pakistan

**Keywords:** intrauterine, orchidectomy, testicular torsion

## Abstract

**Objectives:**

Intrauterine testicular torsion is extremely rare and the exact cause remains largely unknown. It is the result of an ischemic insult intrauterine, which presents as either extra-vaginal or intravaginal testicular torsion. Urgent surgical exploration and fixating the contralateral testis is key in the management of this condition.

**Case presentation:**

We present here the case of a two-day old neonate with in-born right scrotal swelling admitted at Children’s hospital. The patient was born at term via cesarean section at a private hospital. Upon arrival in the emergency department, he was well hydrated, pink at room temperature with good perfusion. Upon examination, the right testis was found to be enlarged, tense, non-tender visibly reddish with overlying skin excoriation. Trans-illumination was negative in right but positive in the contralateral testis. Both hernial orifices were normal. Doppler ultrasound of the inguinoscrotal area found the right testis to be enlarged (15.6*9.4 mm) and showed heterogeneous hypoechoic texture with prominent rete testis and no flow on color doppler analysis. An urgent scrotal exploration was undertaken. Intra-operatively there was frank necrotic right testis with intravaginal torsion of the testis and minimal hydrocele. A right orchidectomy and contralateral orchidopexy were performed.

**Conclusions:**

Intrauterine testicular torsion should be treated as a surgical emergency. We advocate early recognition of intrauterine testicular torsion, alongside surgical exploration and simultaneous contralateral orchidopexy.

## Introduction

Intrauterine torsion of testes was first described by Taylor in 1897 [1] and since then it is widely accepted as a surgical emergency for neonates. Any trauma or ischemic insult that occurs in the fetus during the prenatal stage is termed as intrauterine torsion [2]. Intrauterine torsion of testes is a very rare condition, the incidence of which is estimated to be 6 per 100,000 live births. The most Intrauterine testicular torsion is of extra-vaginal type. The incidence of intravaginal intrauterine testicular torsion is even less than the estimated rate [3]. It was not until 1907 that Rigby and Howard wrote their critically acclaimed paper on Torsion of Testis [4]. Intrauterine testicular torsion is marked by minimal to no discomfort and few localized findings whereas postnatal torsion is an acute manifestation with considerable tenderness and swelling of the testis [5]. Antenatal ultrasound cannot diagnose testicular torsion. It is diagnosed at birth or through routine examination by a neonatologist or a pediatric surgeon. Early diagnosis by pediatric surgeon paramount to early management. This report highlights our experience in the diagnosis and management of intrauterine testicular torsion.

## Case presentation

**Figure 1:**
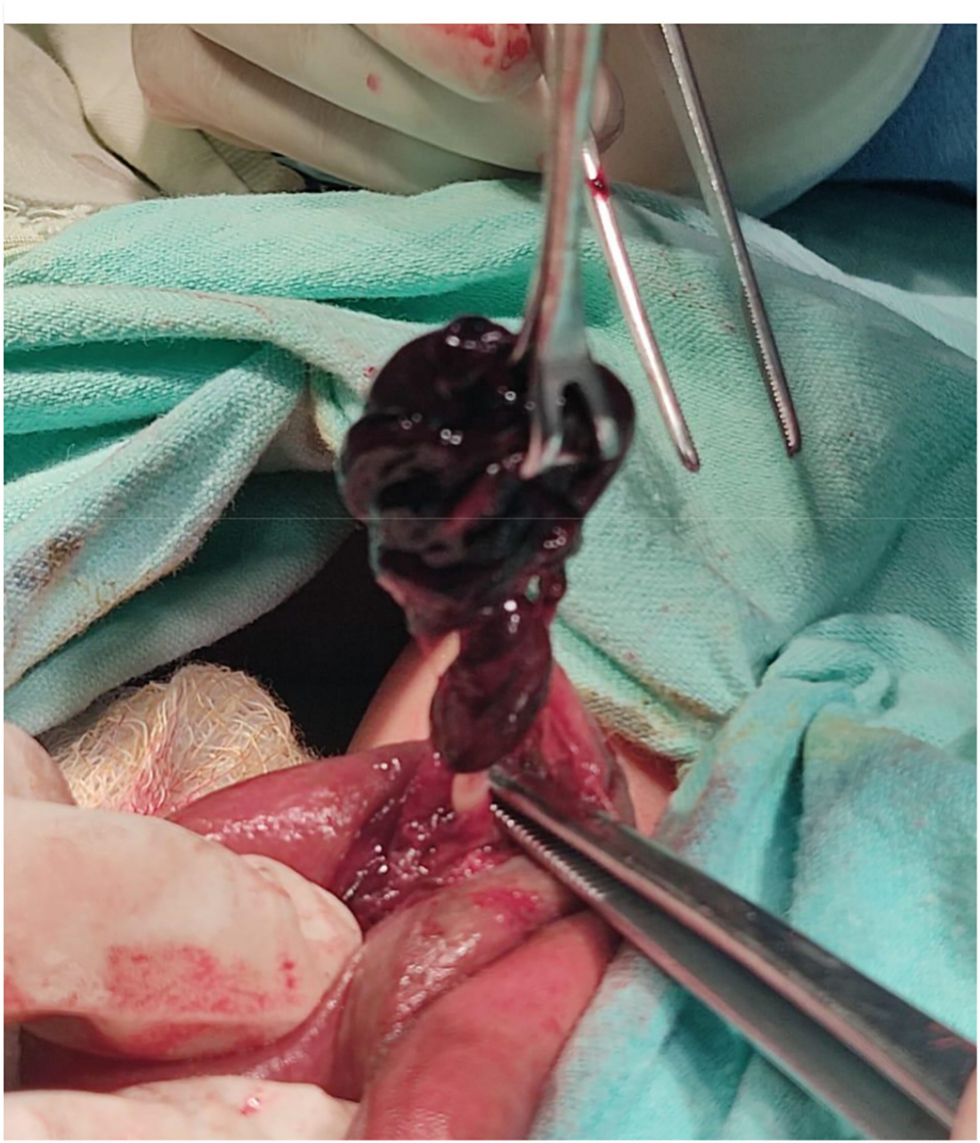
Intraoperatively frank necrotic right testis is shown with intravaginal testicular torsion.

We present here the case of a two-day old neonate with in-born right scrotal swelling admitted at Children’s hospital. The patient was born at term via cesarean section at a private hospital. He was kept in the nursery for one day. The examining doctor referred them for urgent surgical care, but it took them one day to arrive at our hospital. Upon arrival in the emergency department, he was well hydrated, pink at room temperature with good perfusion. Upon examination, the right testis was found to be enlarged, tense, non-tender visibly reddish with overlying skin excoriation. Trans-illumination was negative in the right but positive in the contralateral testis. Both hernial orifices were normal. All the laboratory investigations were performed with an urgent Doppler ultrasound of the inguinoscrotal area. The ultrasound examination found the right testis to be enlarged (15.6*9.4 mm) and showed heterogeneous hypoechoic texture with prominent rete testis and no flow on color Doppler analysis. Left testis appeared normal in size, shape and echotexture with minimal hydrocele. An urgent scrotal exploration was undertaken. Intra-operatively, there was frank necrotic right testis as shown in Figure 1 with intravaginal torsion of the testis with minimal hydrocele. A right orchidectomy and contralateral orchidopexy was then performed.

## Discussion

**Figure 2:**
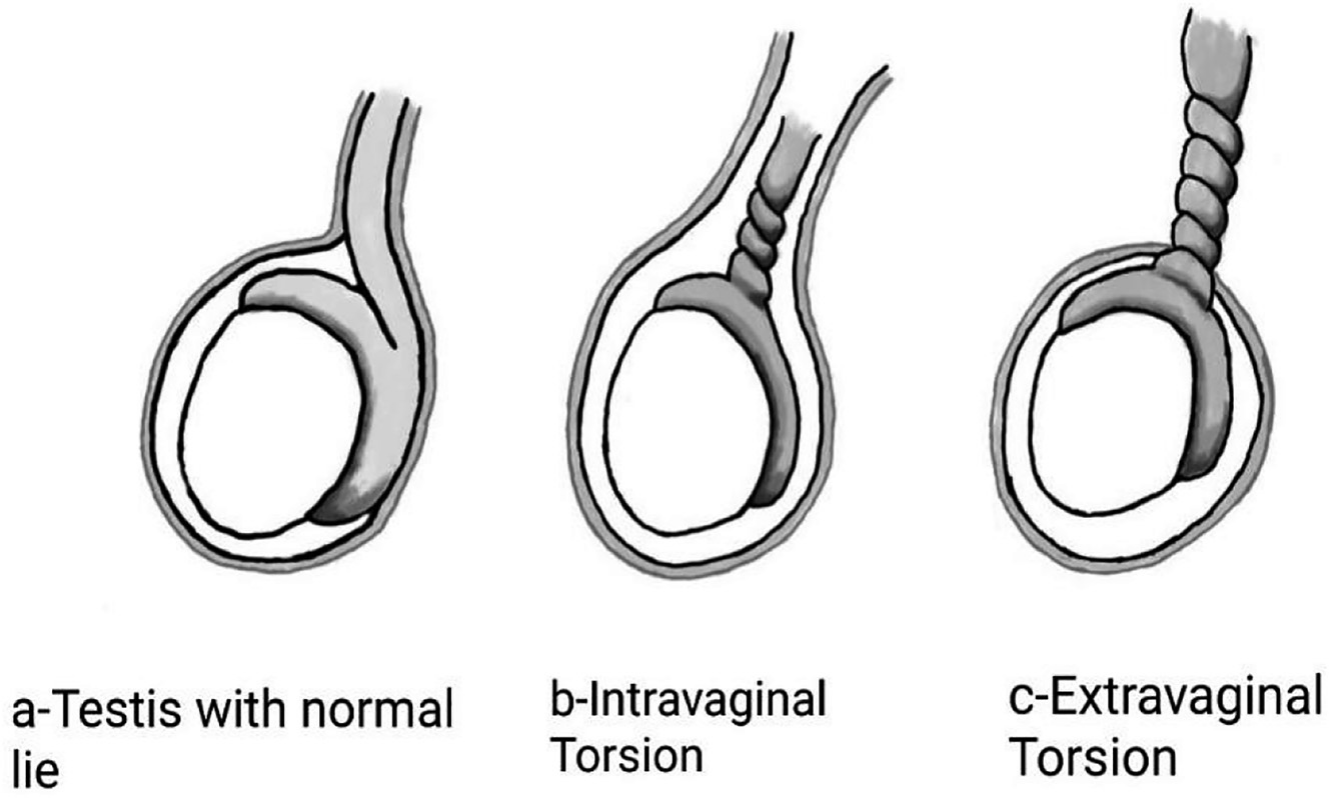
The different types of testicular torsion.

The torsion of the testis occurs due to anatomic anomalies of tunica vaginalis or epididymis that allow excessive testicular mobility inside the scrotum [6]. Testicular torsion is divided into 2 main types based on the anatomy of the tunica vaginalis and the relationship between the testis and the epididymis. Occurrence of intravaginal torsion is mostly prevalent among the prepubescent (13–14 years of age), and while extra-vaginal torsion is the primary mechanism of torsion in fetuses and neonates (Figure 2).

Intrauterine torsion is an example of the extra-vaginal type, though its exact etiology not yet fully known. A widely accredited theory suggests that hypermobility of the tunica vaginalis within the scrotum allows the testis to twist around its sac when there is breech presentation, trauma from labor, or any intrauterine insult [7]. With this type of testicular torsion, both vas and vessels twist resulting in torsion of the testis [8]. This results in ischemic changes such as swelling, degeneration and eventually necrosis and infarction of the testis It is a rare condition, but a newborn can present with it. Thus, adoption of a routine thorough routine examination of all newborns before their discharge from the hospital.

**Figure 3:**
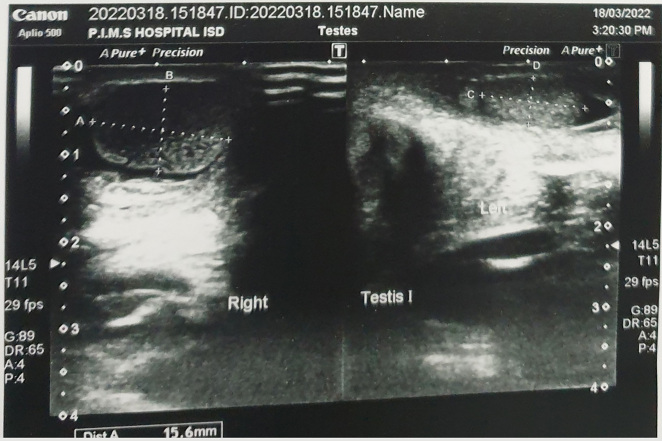
Doppler ultrasound and heterogeneous changes in the right testis.

The diagnosis of intrauterine testicular torsion is clinical, but it is a rare presentation so can easily be missed by clinicians hence should be supported by various diagnostic modalities available [9]. Color Doppler ultrasonography is paramount in establishing differential diagnosis of scrotal anomalies [9, 10]. Since it is an operator dependent modality it can be difficult for a sonologist to pick changes early on. Typically, it would demonstrate enlarged, heterogeneous testis with a hypoechoic central zone confirming necrosis and hyperechoic reflections at the transitional zone in-between the testis parenchyma and tunica albuginea as shown in Figure 3 [6, 9, 11].

The role of Technetium Tc 99m pertechnetate scintigraphy has emerged during the past decade with a sensitivity of 99% and a specificity of 100% [8]. But the color Doppler ultrasound remains the instrument of choice, since the use of same especially by experienced persons is easy to operate, gives reliable results and is relatively cheaper [12].

There has been an ongoing debate on the choice of surgical intervention in the early days of a neonate considering the risk of anesthesia and fact that reduced blood flow to the testis can make them shrunken and even atrophy. Olguner et al. reported a patient at the postnatal 28th hour with right scrotal erythema and swelling. The emergency scan showed hypoperfusion on both sides, and because he underwent surgery immediately, the left testis was adjudged viable, treated with detorsion and saved. We therefore followed the same practice [13].

We conclude that testicular torsion is a recognized surgical emergency and the most common cause of monorchism. In a presentation, one should opt for a complete physical examination, local changes and radiological assessment to preserve the testis, as after just a few hours irreversible changes set in and its difficult to salvage the testis. In the case of the neonate that we attended to, the testis could not be salvaged due to the delay in seeking surgical intervention. To avoid future recurrence, it is recommended that Pediatricians and Neonatologists be vigilant in order to detect this condition early enough for surgical intervention.

## Conclusions

Intrauterine testicular torsion should be treated as a surgical emergency. We advocate early diagnosis of intrauterine testicular torsion, alongside surgical exploration and simultaneous contralateral orchidopexy.
